# Dengue Mosaic Vaccines Enhance Cellular Immunity and Expand the Breadth of Neutralizing Antibody Against All Four Serotypes of Dengue Viruses in Mice

**DOI:** 10.3389/fimmu.2019.01429

**Published:** 2019-06-20

**Authors:** Jue Hou, Shubham Shrivastava, Christopher C. Fraser, Hooi Linn Loo, Lan Hiong Wong, Victor Ho, Katja Fink, Eng Eong Ooi, Jianzhu Chen

**Affiliations:** ^1^Interdisciplinary Research Group in Infectious Diseases, Singapore-MIT Alliance for Research and Technology, Singapore, Singapore; ^2^Singapore Immunology Network, A^*^Star, Singapore, Singapore; ^3^Emerging Infectious Diseases Program, Duke-NUS Graduate Medical School, Singapore, Singapore; ^4^Koch Institute for Integrative Cancer Research and Departments of Biology, Massachusetts Institute of Technology, Cambridge, MA, United States

**Keywords:** dengue viruses, mosaic vaccine, T cell responses, neutralizing antibodies, cross-reactivity

## Abstract

An estimated 400 million people in the world are infected with any of the four types of dengue virus (DENV) annually. The only licensed dengue vaccine cannot effectively prevent infection with all of the four DENVs, especially in those immunologically naïve at baseline. In this study, we explored a mosaic vaccine approach, which utilizes an artificial recombinant sequence for each serotype to achieve maximum coverage of variant epitopes in the four DENVs. We determined the immunogenicity and cross-reactivity of DNA plasmids encoding individual mosaic sequences or the natural sequences in mice. We show that the mosaic vaccines, particularly those targeting DENV serotype 1 and 2, improved vaccine immunogenicity by increasing the percentage of antigen-specific IFNγ- or TNFα-secreting CD4 and CD8 T cells, and titers of neutralizing antibodies. The mosaic vaccine diversified and broadened anti-dengue T cell responses and cross-reactive neutralizing antibodies against all four serotypes. The mosaic vaccines also induced higher level of antigen-specific B cell responses. These results suggest that mosaic vaccines comprising of DENV serotype 1 and 2 variant epitopes could stimulate strong and broad immune responses against all four serotypes.

## Introduction

Dengue is a mosquito-borne viral disease caused by dengue viruses (DENV). DENVs are a single stranded, positive sense RNA virus belonging to the flavivirus genus of the flaviviridae family (1, 2). There are four antigenically distinct, but closely related types of DENV (3, 4). Based on the viral envelope (Env) protein amino acid sequences, these four serotypes share 60–80% homology (5, 6). Exposure to any one of the four DENVs induces life-long, type-specific immunity. However, cross-reactive immunity to the other DENVs is only partial and short-lived (7–10). Subsequent infections with a different DENV may instead result in increased risk of severe dengue, which is characterized by hypovolemic shock from vascular leak, internal hemorrhage or organ dysfunction. This increased risk of severe disease during secondary DENV infection could be contributed, at least in part, by antibody-dependent enhancement (ADE) (11), by which cross-reactive non- or sub-neutralizing levels of antibodies induced from the first infection bind heterotypic DENV to enable virus entry into target cells via Fc-gamma receptors (FcγR) (12–14). Other factors, such as viral factors and host genetic susceptibility also contribute toward dengue pathogenesis (15). Consequently, a dengue vaccine that can prevent infection with all four DENVs remain a public health need.

Efforts to develop safe and effective dengue vaccines are presently at various stages of progress. Six vaccine candidates are currently in clinical trials, including live-attenuated viruses (16, 17), recombinant proteins (18), DNA (19–21), and inactivated viruses (22). Additional candidates, such as virus-vectored and virus like particle (VLP)-based vaccines (23, 24), are under pre-clinical evaluation. To date, only a live attenuated tetravalent vaccine, Dengvaxia (CYD-TDV), has been approved in a limited number of countries. Although Dengvaxia exhibited good protection against DENV3 (~80%) and DENV4 (~90%), the protection was 50–60% for DENV1 and 35–59% for DENV2 (25–27). These type-specific vaccine efficacies were observed despite similar type-specific geometric mean virus neutralization titers from Dengvaxia vaccination (25). Moreover, follow up studies have since suggested increased risk of hospitalized dengue among vaccinated children who were seronegative at baseline compared to the placebo arm. Consequently, the World Health Organization (WHO) has recommended that this vaccine should only be used in people who had prior dengue infection. There is thus an urgent need to develop vaccines that can stimulate strong protective immunity against all four DENV serotypes.

A potential limitation in the current pipeline of dengue vaccine candidates is the assumption that immunity generated by each of the four vaccine strains would protect against all DENVs despite the genetic heterogeneity within each type of DENV. Indeed, recent studies raise the possibility that re-infection with the same type of DENV is possible possibly due to antigenic variation. To address this possible limitation, we explored in this study a “Mosaic Vaccine” approach (28, 29). This approach relies on *in silico* algorithms to select vaccine sequences to include the maximal diversity of potential T cell epitopes from the natural sequences to more closely match and maximally represent the sequence of natural virus strains (29). The mosaic strategy has been applied to development of vaccines against HIV (30, 31), HCV (32), influenza virus (33), and bovine viral diarrhea virus (34). The mosaic vaccine augments breadth and depth of the HIV antigen-specific T cell responses in monkeys (30) and human (35). Here, we designed, constructed and evaluated four mosaic vaccines using the precursor membrane (prM) and envelope (Env) gene sequences from each DENV serotype in a DNA vaccine formulation. Our results indicate that the mosaic DENV1 and DENV2 DNA vaccine approach improves both the homotypic and heterotypic cellular and humoral immune responses to all four DENV serotypes.

## Materials and Methods

### Mosaic Vaccine Design

Three thousand three hundred and forty five sequences were collected from ViPR database (as of September 2015) that included all four serotype dengue virus strains with full length prM and Env sequences. The collected sequences were submitted to an online mosaic vaccine designer (https://www.hiv.lanl.gov/content/sequence/MOSAIC/makeVaccine.html) to generate four mosaic sequences, one for each DENV serotype. Wild type sequences from four clinical dengue strains (DENV1/2402DK1, GenBank: EU081230.1; DENV2/3295DK1, GenBank: EU081177.1; DENV3/863DK1, GenBank: EU081190.1; and DENV4/2270DK1, GenBank: GQ398256.1), which were isolated from dengue cases in Singapore (36), were selected as controls.

### Construction and Production of DNA Vaccines

Four mosaic DNA sequences and four wild type DNA sequences were synthesized using humanized codons and cloned into NTC7482vector (Nature Technology) (37) under the control of an optimized chimeric promoter SV40-CMV-HTLV-1 and a bovine growth hormone polyadenylation signal. A consensus Kozak sequence was added at −6 nucleotides to maximize protein translation. The DNA plasmids were prepared by Endotoxin-free Giga Plasmid Kit (Qiagen). All DNA vaccines were aliquot and stored at −80°C until use.

### Mice and Immunization

Eight-week-old female C57BL/6J mice were used for all experiments. Mice were bred and housed at the Animal Facility, National University of Singapore (NUS). To investigate immunogenicity of individual mosaic candidate of each serotype (labeled as pMosaic 1–4), 10 mice per group were immunized with 3 doses of 50 μg plasmid DNA intramuscularly, 2 weeks apart. In parallel, mice were immunized with plasmid DNA containing wild type sequences of each serotype (named as pDengue 1–4). Table 1 lists plasmids and abbreviations. All animal procedures and care were approved by the NUS Research Ethics Committee.

**Table 1:** The annotations of plasmids.

**Plasmid**	**Type**	**Serotype**	**Abbreviation**
pDengue 1	Wild type	Serotype 1	pDen1
pDengue 2	Wild type	Serotype 2	pDen2
pDengue 3	Wild type	Serotype 3	pDen3
pDengue 4	Wild type	Serotype 4	pDen4
pMosaic 1	Mosaic	Serotype 1	pMos1
pMosaic 2	Mosaic	Serotype 2	pMos2
pMosaic 3	Mosaic	Serotype 3	pMos3
pMosaic 4	Mosaic	Serotype 4	pMos4

### Intracellular Cytokine Staining

Cytokine production by splenocytes from immunized mice was assessed by intracellular cytokine staining as described previously (38). Briefly, 1 million splenocytes were stimulated with prM and Env peptide cocktails (one prM peptide [RALIFILL] and two Env peptides [MTMRCIGI and VSWTMKIL]) that were previously demonstrated to be dominant epitopes (39) (final concentration of each peptide was 5 μg/ml) for each DENV serotype or mock control for 6 h at 37°C in the presence of Brefeldin A (Golgi plug, BD Biosciences). Cells were surface stained with anti-CD3 (Clone 145-2C11), anti-CD4 (Clone RM4-5), anti-CD8 (Clone 53-6.7) antibodies, and viability dye FSV780 (BD Biosciences) on ice for 30 min. Cells were fixed and permeabilized with Fix/Perm buffer (BD Biosciences) for 30 min at 4°C in the dark and then incubated with anti-IL2 (Clone JES6-5H4), anti-TNFα (Clone MP6-XT22) and anti-IFNγ (Clone XMG1.2) monoclonal antibodies. Samples were acquired on an LSR II flow cytometer (BD Biosciences) and data analyzed using FlowJo version 9.5.2 (Tree Star).

### ELISA Assay

The levels of DENV-specific antibodies were assessed by serotype specific ELISAs (40). Recombinant EDIII was produced in *E. coli in-house* and Env protein of each serotype was purchased from CTK Biotech. 96-well plates were coated with 1 μg/ml protein and kept at 4°C overnight. The next day, plates were washed 5 times with PBST (0.05% Tween 20) and blocked with 5% BSA at 4°C overnight. After washing, serum samples were added to plates in dilution from 1:200 to 1:25600 and incubated for 2 h in 37°C. Secondary HRP-labeled anti-mouse IgG diluted to 1:5000 was added to plates and incubated for 1 h at 37°C. TMB substrate was added and the absorbance was read at 450 nm. The cut-off threshold was set at least two times higher than the result of negative sera sample. The titer was determined by the last dilution giving value above the cut-off threshold.

### Dengue Plaque Reduction Neutralization Test (PRNT)

Neutralizing antibody (nAb) titer was determined by PRNT as previously described (41, 42). Briefly, mouse sera were inactivated at 56°C for 30 min and serially diluted with RPMI-1640 supplemented with 2% FBS. Diluted sera were mixed with equal volume of one target virus (30–50 PFU/well): DENV1/2402DK1, DENV2/3295DK1, DENV3/863DK1, or DENV4/2270DK1, and incubated at 37°C for 1 h. The mixtures were transferred onto a monolayer of BHK21 cells and allowed to absorb for 1 h at 37°C. Cells were overlaid with 1% CMC with 2% FBS, antibiotics, and NaHCO_3_. After 6 to 7 days of incubation at 37°C, 5% CO_2_, the CMC layer was removed and fixed in 7.5% formalin for 1 h. After removal of formalin and wash, the cell layers were fixed by running tap water and stained with 1% crystal violet solution for 1 h. The plates were then washed in water and air dried. The plaques were counted. The highest serum dilution that resulted in 50% or more reduction of the average number of plaques as compared to the virus control wells was considered as the neutralizing endpoint titer (PRNT_50_).

### B Cell Assays

To identify antigen-specific B cells, Env proteins (CTK Biotech) were conjugated with Alexa Fluor dye. The recombination Dengue 1 and 2 Env proteins (~50 kDa) were cloned from DENV1/VN/BID-V949/2007 and DENV2/GWL39 IND-01 strains, respectively. The proteins with deletion of C-terminus transmembrane domains were expressed in the *Drosophila* S2 insect cell line and purified up to 95% purity. The recombinant Env proteins were then conjugated with Alexa Fluor 647 (AF647) and Alexa Fluor 548 (AF548), respectively, by protein labeling kits (Thermo Fisher Scientific). One million splenocytes were incubated with DENV1/Env-AF647 and DENV2/Env-AF548 probes on ice for 30 min in the dark. The cells were surface stained with fluorescence conjugated anti-CD90.2, anti-F4/80 (Clone BM8), anti-CD11c (Clone N418), anti-CD4 (Clone GK1.5), anti-CD8 (Clone 53-6.7), anti-Ly6G (Clone RB6-8C5), anti-NK1.1 (Clone PK136), anti-IgM (Clone R6-60.2), anti-IgD (Clone 11-26c), anti-GL7 (Clone GL7), anti-CD45R (Clone RA3-6B2), anti-CD38 (Clone 90), and FSV780 (BD Bioscience) on ice for 30 min. For the intracellular staining, cells were fixed and permeabilized with Phosflow Lyse/Fix buffer (BD Bioscience) for 10 min at 37°C in the dark and subsequently Phosflow Perm/Wash buffer for 30 min at room temperature. Cells were incubated with anti-Ig_(H+L)_ and anti-Bcl6 (Clone K112-91) for 30 min at 4°C in the dark. Samples were acquired on an X20 flow cytometer (BD Biosciences) and data analyzed using FlowJo version 9.5.2 (Tree Star).

### Statistical Analysis

Statistical analysis was performed using the Mann-Whitney *U*-test with Prism 7.0 software (GraphPad Software Inc.) or R (version 3.4.3) to compare paired mosaic vaccine and wild-type vaccine.

## Results

### Mosaic Sequences Have a Higher Epitope Coverage Than Wild-Type Sequences

**Figure 1:**
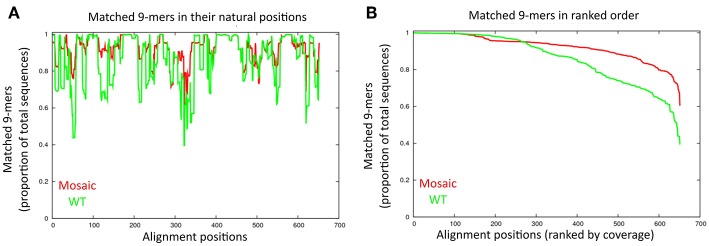
Comparison of amino acid sequences between the mosaic sequences and wild type sequences. **(A)** Matched 9-mers (epitopes) are shown by alignment position. The proportion of 9-mers epitopes in the set of collected dengue sequences that are exactly matched by 9-mers in the mosaic and wild-type sequences are across the alignment. Each x-value represents a column in the test set alignment; the y-values represent the proportions of the total input sequences. The red and green line indicate the mosaic sequences and wild-type sequences, respectively. **(B)** Proportion of 9-mers in the test set that are exactly matched in the antigen sets of mosaic and wild-type across the alignment. Each x-value represents a column in the test set alignment, sorted by y-value (descending); the y-values represent the proportion of the total input sequences.

Each of the four DENVs is composed of multiple different genotypes with significant sequence diversity in the prM and Env proteins. We thus used the Mosaic Vaccine Designer (28, 29) to obtain maximum coverage of potential T cell epitopes in prM and Env across diverse sequences in each serotype. The mosaic sequences were designed from a set of reference prM and Env sequences using a genetic algorithm to maximize potential epitope coverage of highly diverse homologous antigens. We obtained four mosaic prM and Env sequences, one for each serotype (Supplement Figure 1). By comparing the mosaic prM and Env sequences to their respective wild type sequences, the mosaic sequences had a higher coverage of T cell epitopes than wild type sequences at most of the positions (Figures 1A,B). For example, the mosaic sequences showed higher epitope coverages than the wild-type sequences at the amino acid positions 40–60, 110–150, 200–250, and 320–350 (Figure 1A). At the ranked 600 aligned positions, the coverage was up to 80% for the mosaic sequences compared to 60% in the wild type sequences (Figure 1B).

### The pMos1 and pMos2 Stimulate Stronger Homotypic and Heterotypic T Cell Responses

To produce DNA vaccines, we cloned the four mosaic sequences and their corresponding representative wild type sequences into DNA vaccine vector NTC4782. The resulting plasmids are referred to as pMos1 to pMos4 (for mosaic sequences) and pDen1 to pDen4 (for wild type sequences) (Table 1). In this vector, the expression of antigen gene is driven by an optimized chimeric promoter SV40-CMV-HTLV-1, which has been shown to drive significantly higher expression than the traditional human cytomegalovirus (CMV) promoter (43, 44). In addition, the encoded protein is shuttled into the secretory pathway using an optimized tissue plasminogen activator (TPA) signal peptide to stimulate humoral immune responses (45, 46).

**Figure 2:**
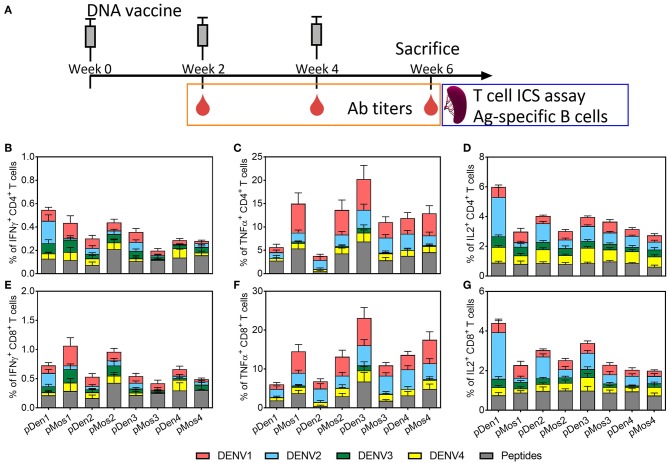
Comparison of T cell responses induced by mosaic and non-mosaic vaccines. **(A)** Schematic diagram of experimental protocol. C57BL/6 mice were inoculated with three doses of DNA plasmid intramuscularly every 2 weeks. Mice were bled 2 weeks after each immunization, and sacrificed 2 weeks after the third immunization. Splenocytes were used for assaying cytokine expression by CD4 and CD8 T cells following ex vivo stimulation with DENV or peptides. **(B–G)** Comparison of the average percentages of CD4 or CD8 T cells that express IFNγ, TNFα, and IL2. Vaccines used for immunization are labeled on the x-axis; and the ex vivo stimulating antigens are indicted by different colors. N = 10 mice per group.

To compare the immunogenicity of mosaic vaccines and their corresponding wild type vaccines, we immunized C57BL/6J mice with pMos or pDen plasmids intramuscularly at 2-weeks interval for three times (Figure 2A). Mice were bled 2 weeks after each immunization and sacrificed 2 weeks after the third immunization. Splenocytes were stimulated *ex vivo* with four serotype viruses individually and one peptide pool (containing one peptide from prM and two peptides from the Env) (39) as shown in Table 2. Expression of IFNγ, TNFα, and IL2 by CD4 and CD8 T cells were quantified by intracellular staining and flow cytometry (Supplement Figure 2) and mock immunization data provided in Supplement Figure 3.

**Table 2:** The amino acid sequence comparison between peptides used for ICS assay and the corresponding epitopes in each vaccine.

	**M60-67 (K^**b**^ 8-mer)**	**E2-6 (K^**b**^ 8-mer)**	**E451-458 (K^**b**^ 8-mer)**
Peptides used	RALIFILL	MTMRCIGI	VSWTMKIL
pMos1	**KGI**IFILL	M**A**MRC**V**GI	VSW**I**MKI**G**
pDen1	**KGI**IFILL	M**A**MRC**V**GI	VSWTMKI**G**
pMos2	RALIFILL	MTMRCIGI	VSWTMKIL
pDen2	R**V**LIFILL	MTMRCIGI	VSWTMKIL
pMos3	**KVV**IFILL	MTMRC**V**GI	VSWTMKI**G**
pDen3	**KVV**IFILL	MTMRC**V**GI	VSW**V**MKI**G**
pMos4	R**V**LIFILL	M**A**MRC**V**GI	VSW**V**MKI**G**
pDen4	R**TVF**F**V**LM	**YG**MRC**V**GI	VSW**MVR**IL

*The amino acids differ from the reference sequences were annotated as bold*.

The immunization with pMos1 and pMos2 enhanced homotypic CD4 and CD8 T cell responses with serotype specific or peptide stimulations than immunization with pDen1 and pDen2, respectively. For example, the mean value of the percentages of CD4 and CD8 T cells expressing IFNγ were ~2 and ~3 times higher, respectively, from pMos1- than pDen1-immunized mice following stimulation with DENV1 virus (Figures 2B,E). The percentages of CD4 (mean value 14.9 vs. 5.6%, *p* = 0.053) and CD8 (mean value 14.5% vs. 6%, *p* = 0.046) T cells expressing TNFα were ~5 times higher (Figures 2C,F) for pMos1 compared to pDen1 following DENV1 stimulation. Moreover, pMos2 immunization induced higher percentages of TNFα-expressing CD4 (Figures 2B,C) and CD8 (Figures 2E,F) T cells than pDen2 immunization (*p* = 0.007 and 0.012, respectively) when splenocytes were stimulated with the consensus peptide pool.

The pMos1 and pMos2 immunization also induced stronger heterotypic CD4 and CD8 T cell responses. For example, the percentages of CD4 and CD8 T cells expressing TNFα were ~4 and ~3 times higher, respectively, in pMos2 than pDen2 immunized mice following stimulation with DENV1 virus (Figures 2C,F, *p* = 0.029 and 0.044, respectively). Interestedly, pMos1 reduced the anti-DENV2 heterotypic CD4 (Figure 2B) and CD8 (Figure 2E) T cell responses as indicated by decreased IFNγ expression. There was no significant enhancement in the percentages of cytokine expressing CD4 and CD8 T cells following immunization with pMos3 and pMos4 compared with pDen3 and pDen4, respectively (Supplementary Table 1). The mosaic vaccine of each serotype did not improve the IL2 secretion by CD4 and CD8 T cells (Supplementary Table 1). However, the pDen1 and pDen2 vaccines induced significantly higher IL2 expression in CD4 (*p* = 0.0003 for pDen1 vs. pMos1, *p* = 0.001 for pDen2 vs. pMos2), and CD8 (*p* = 0.0005 for pDen1 vs. pMos1, *p* = 0.01 for pDen2 vs. pMos2) T cells following stimulation with DENV2 virus as compared with pMos1 and pMos2 vaccines, respectively.

Together, these results suggest that pMos1 and pMos2 immunization, but not pMos3 and pMos4 immunization, appears to induce stronger homotypic T cell response and broaden the heterotypic T cell responses.

### Mosaic DNA Vaccines Broaden Cross-Reactive Neutralizing Antibody Responses

**Figure 3:**
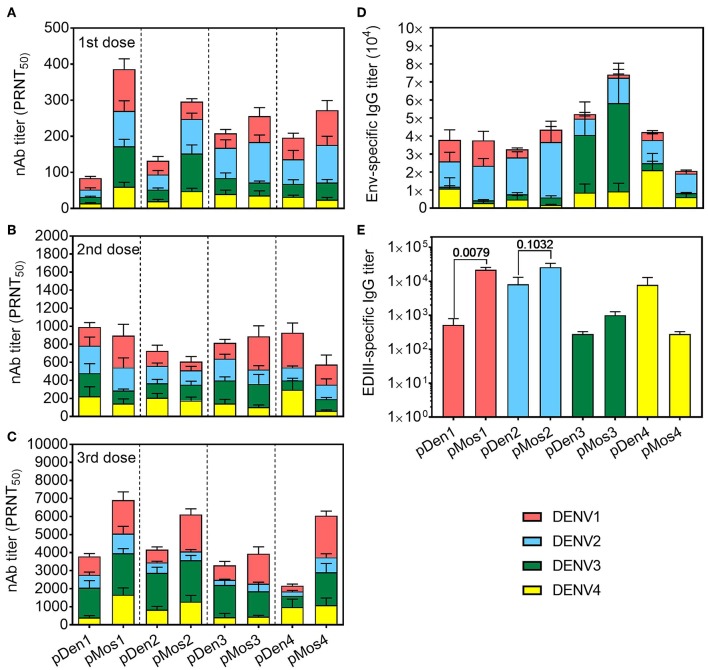
Mosaic vaccines induced stronger and more cross-reactive nAb responses. *C57BL/6* mice were inoculated with three doses of DNA plasmid intramuscularly every 2 weeks. Mice were bled 2 weeks after each immunization, and sera were used for assaying antibody responses. **(A–C)** Neutralizing Ab titers in the sera after the 1st **(A)**, 2nd **(B)**, and 3rd **(C)** immunizations were determined by PRNT assay. Four DENV serotypes were used to determine the cross-reactivity of nAbs. PRNT_50_ was the Ab dilution that gave 50% reduction of the average number of plaques. **(D,E)** IgG Ab titers in the sera after the 3rd immunization specific for Env protein **(D)** and EDIII **(E)** as measured by ELISA. The data shown are mean ± SEM (*n* = 10 per group).

We determined the induction of neutralizing antibodies (nAb) 2 weeks after each immunization by dengue plaque reduction neutralization test (PRNT). As expected, the levels of nAb increased with each immunization (Figures 3A–C). Overall, immunization with pMos DNA vaccines induced higher titers of nAb than pDen DNA vaccines, especially after the first and the third immunization. Strikingly, mosaic vaccines pMos2 induced higher titers of cross-reactive nAb against DENV3 and 4 post the 1st and against DENV1 after the 3rd immunization. For example, all four pMos vaccines induced higher titers of anti-DENV1 nAbs than immunization with corresponding pDen vaccines after the 3rd dose, although the differences between pMos1 and pDen1 or between pMos3 and pDen3 were not statistical significance (Figure 3C). pMos1 and pMos2 vaccines induced higher homotypic and heterotypic nAb than pDen1 and pDen2 vaccines, respectively, after the 1st dose. Although some comparisons did not reach statistical significance, e.g., pMos1 vs. pDen1 with DENV1 stimulation after the 1st dose (*p* = 0.08), the mean PRNT values were dramatically elevated in the mice immunized with pMos1 compared with pDen1 immunized mice (386 vs. 84 with DENV1, 270 vs. 32 with DENV2, 172 vs. 32 with DENV3 and 60 vs. 14 with DENV4 stimulation). After the 3rd dose, the pMos2 developed nAb cross-reactive with DENV1, and the pMos1 induced nAb cross-reactive with DENV4 virus.

We also measured the total IgG Ab titers specific for Env protein and domain III of Env (EDIII) of all four serotypes. Each serotype vaccine tended to generate stronger homotypic than heterotypic antibody responses, i.e., both pDen1 and pMos1 induced higher proportion of anti-DENV1 Ab than the other serotype vaccines (Figure 3D). But all of them induced high titers of IgG to DENV2 Env. Furthermore, the pMos1 vaccine induced significantly higher titers of anti-EDIII antibodies than pDen1 vaccine (Figure 3E). The mean values of anti-EDIII titer were 21,000 for pMos1 vs. 500 for pDen1. The pMos2 vaccine also enhanced the anti-EDIII titer compared with pDen2 vaccine (mean value 26,000 vs. 8,200). In contrast, no difference in anti-EDIII titer was detected between pMos3 and pDen3 or between pMos4 and pDen4. These results suggest that the mosaic vaccines, especially pMos1 and pMos2, induce stronger nAb responses and broaden the cross-reactivity of nAbs.

### The pMos1 and pMos2 Vaccines Stimulate Cross-Reactive Memory B Cell Responses and Bcl-6 Expression

**Figure 4:**
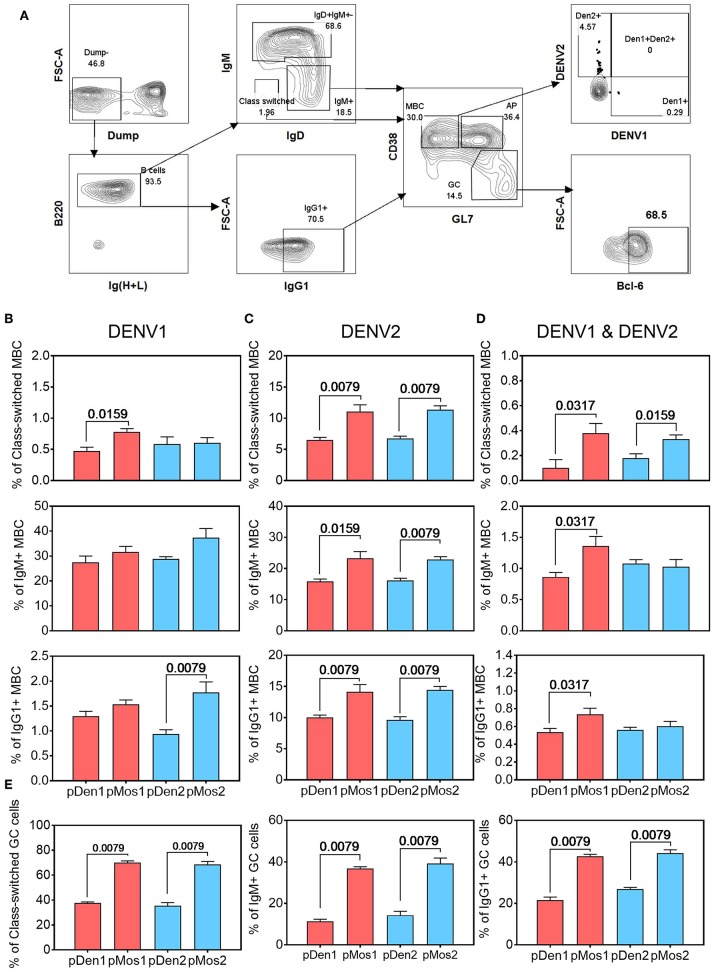
pMos1 and pMos2 promote antigen-specific cross-reactive B cell responses. C57BL/6 mice were inoculated with three doses of DNA plasmid intramuscularly every 2 weeks. Mice were sacrificed 2 weeks after the third immunization. Splenocytes were stained with fluorescence conjugated DENV1 and DENV2 Env proteins plus antibodies specific for different B cell surface markers. **(A)** Representative flow cytometry plots showing gating strategies to identify antigen-specific B cells that are class-switched, IgM^+^ or IgG^+^. The CD90.2, F4/80, CD11c, CD4, CD8, Ly6G, and NK1.1 antibodies were used for dump gating. The MBC refer to memory B cells, the GC refer to germinal center, and AP refer to activated precursors. Comparison of percentages of DENV1 Env-reactive **(B)**, DENV2 Env-reactive **(C)**, and cross-reactive **(D)** B cells that are class-switched (top row), IgM^+^ (middle row) and IgG1^+^ (bottom row). **(E)** Comparison of percentages of the class-switched, IgM^+^ and IgG1^+^ B cells that express Bcl-6 in splenocytes of mice immunized with pDen1, pMos1, pDen2, or pMos2. The data are mean ± SEM (n = 10 per group). The statistical comparison was assessed by Mann-Whitney U test.

We labeled DENV1 and 2 Env proteins with different Alexa Flour dyes and used them to identify antigen-specific B cell subsets. B cells were divided into class-switched (IgM^−^IgD^−^), IgM^+^ and IgG1^+^ B cells; and the latter were further divided into germinal center (CD38^−^GL7^+^) and memory B cells (CD38^+^GL7^−^) subsets (Figure 4A). The pMos1 plasmid immunization stimulated a higher percentage of DENV1 Env-reactive class-switched B cells than pDen1 plasmid immunization (Figure 4B, top panel). Additionally, pMos1 also induced higher percentages of DENV2 Env-specific and DENV1^+^DENV2^+^ Env-specific class-switched (top panel), IgM^+^ (middle panel) and IgG1^+^ (bottom panel) B cells than pDen1 immunization (Figures 4C,D). The pMos2 plasmid immunization induced higher percentages of DENV2 Env-reactive B cells than pDen2 plasmid immunization (Figure 4C), without enhancing heterotypic B cell responses (DENV1^+^DENV2^+^), except class-switched memory B cells (Figure 4D). These results suggest that pMos1 induces stronger heterotypic B cells responses, but pMos2 immunization trends to enhance homotypic B cell responses.

We also measured expression of transcriptional factor Bcl-6 in germinal center B cell subsets, because it is required for B cell activation, proliferation and differentiation. As expected, Bcl-6 expression was higher in germinal center of the class-switched, IgM^+^ and IgG1^+^ B cell subsets from pMos1 and pMos2 immunized mice than from pDen1 and pDen2 immunized mice (Figure 4E). Thus, enhanced Bcl-6 expression on B cell subsets in the germinal center could have contributed to stronger B cell responses after pMos1 and pMos2 immunization.

## Discussion

Stimulating potent immune responses against DENV1 and DENV2 serotypes has been a challenge in Dengvaxia development. Although Dengvaxia confers good protection against DENV3 and DENV4, protection against DENV2 is poor. In this study, we investigated cellular and humoral immune responses generated by serotype specific natural and mosaic DNA vaccines in mice. The pMos1 and pMos2 were significantly more effective than pDen1 and pDen2, respectively, in inducing stronger and broader T cell and antibody responses. Higher percentages of CD4 and CD8 T cells expressed IFNγ and TNFα following both homotypic and heterotypic stimulations. Similarly, higher titers of homotypic and heterotypic nAbs were induced. The pMos1 and pMos2 also induced higher titers of EDIII-reactive Abs. The enhanced Ab responses were correlated with the presence of class-switched, IgM^+^ and IgG1^+^ memory B cells that expressed Bcl-6. These results suggest that epitope-optimized mosaic sequences can elicit higher and broader cellular and humoral immune responses *in vivo* and therefore provide an approach to enhance immune responses against DENV1 and DENV2.

We detected high titers of DENV2 Env-reactive Abs in all immunizations, however, DENV2-specific nAb titers were lowest (Figures 3 C,D). Similarly, pMos2 induced high titers of DENV2 EDIII-reactive Abs, but the titer of DENV2 nAb was low. Our results are consistent with previous studies showing that the titers of nAbs are not proportional to total Env-reactive or EDIII-reactive Ab titers. The poor induction of DENV2 nAb by mosaic DNA vaccines is also consistent with the poor induction of nAb by attenuated DENV2 virus in Dengvaxia. It is notable that mosaic vaccines were designed by maximize the CD8 T cell epitopes, but they still induced nAb responses. This is likely due to the high sequence identity/homology between the mosaic and wild type sequences.

However, the mosaic approach did not significantly improve the immunogenicity of pMos3 and pMos4 candidates. Based on the T cell response (e.g., TNFα expressed by CD4 and CD8 T cells, Figures 2C,F) and nAb titers (Figure 3), the wild type pDen3 and pDen4 have the abilities of generating comparable homotypic immunity as pMos3 and pMos4 did. Therefore, the improvements on the pDen3 and pDen4 through the mosaic approach were limited. Even though, we still find the pMos4 enhanced the homo- and heterotypic nAbs titers after the 3rd immunization (Figure 3C). Moreover, based on the clinical trial outcomes of licensed Dengvaxia (47) vaccine and vaccines TV003/005 (48) and TDV (49) that were in clinical trials, they are able to either provide high efficacy or induce high nAbs/seroconversion against DENV3 and DENV4. It seems that induction of sufficient antibody responses to DENV3 and DENV4 rather than DENV1 and DENV2 is easier. Therefore, further improving pMos1 and pMos2 could help to overcome the challenge of inducing strong protection immune responses to DENV-1 and DENV2 infections.

The mosaic strategy strikes to globally modify antigenic epitopes. The pMos1 and pMos2 vaccines improve CD4 and CD8 T cell responses (Figure 2) by peptide stimulation compared to the pDen1 and pDen2 vaccine, respectively. According to the epitope alignments (Table 2), the mosaic and wild-type vaccines were highly matching on the corresponding epitopes but slightly differ from the peptide sequence used in *ex vivo* ICS assay. For instance, even though the epitope similarities were high with only one amino acid variance in E451-458 peptide between the wild type and mosaic DNA vaccines, but only pMos1 not pDen1 was able to induce higher T cells responses. This observation would indicate that the elevated T cell responses induced by mosaic vaccine could be induced by the known epitopes as well as unknown epitopes. Further studies involving epitope mapping are required to fully understand the observed benefits of mosaic DENV vaccines.

Mosaic antigens represent a potential strategy to improve humoral and cellular immune responses against diverse dengue serotypes and genotypes. It appears that T cell responses were improved with mosaic sequences compared to natural sequences. Given this observation, the DNA vaccine design could be expanded to include NS3 and NS5 proteins, which are known to induce efficient T cell responses. The capacity of the vaccine to induce neutralizing antibodies, particularly to DENV2, needs to be improved. The improved germinal center and memory B cell responses for pMos1 and pMos2 compared to pDen1 and pDen2 are promising and warrant further investigation. Overall, the mosaic vaccine strategy as a proof-of-concept offers an opportunity to overcome the hurdle of producing sufficient anti-DENV1/DENV2 immunity.

## Data Availability

All datasets generated for this study are included in the manuscript and/or the Supplementary Files.

## Ethics Statement

This study was carried out in accordance with the recommendations of NUS Research Ethics Committee. The protocol was approved by the NUS Research Ethics Committee.

## Author Contributions

JH and JC designed this study and drafted the manuscript. JH, HL, and LW conducted the T cell assay. JH and HL conducted the nAb assay. SS and VH carried out ELISA assay. SS, CF, KF, and EO reviewed and revised the manuscript.

### Conflict of Interest Statement

The authors declare that the research was conducted in the absence of any commercial or financial relationships that could be construed as a potential conflict of interest.
