# Supplementary material for: Dengue Mosaic Vaccines Enhance Cellular Immunity and Expand the Breadth of Neutralizing Antibody Against All Four Serotypes of Dengue Viruses in Mice
Source: Front Immunol. 2019 Jun 20;10:1429. doi: 10.3389/fimmu.2019.01429 (PMC6596366; doi:10.3389/fimmu.2019.01429)
Supplement: Supplementary file 3 [file Data_Sheet_2.PDF]

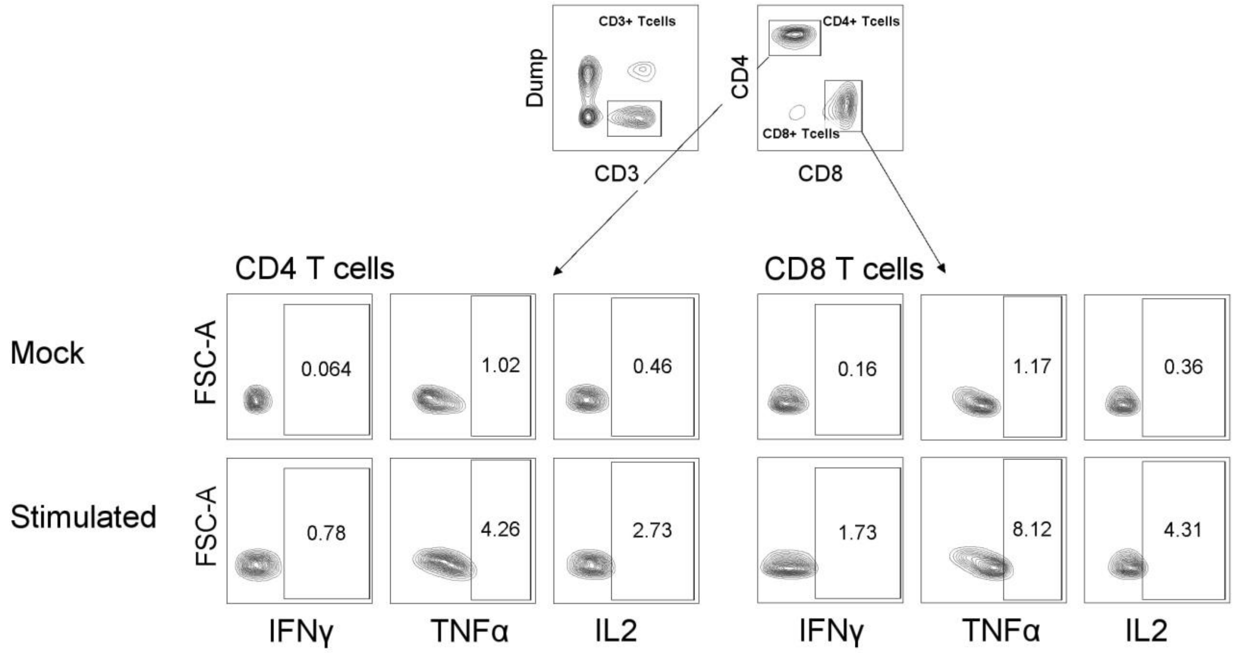


# Supplement Figure 2. Flow cytometry gating strategy on T cells.

Representative flow cytometry analysis comparing IFNγ, TNFα and IL2 expression in CD4 and CD8 T cells with or without DEN1 stimulation of splenocytes from a pMos1-immunized mouse. The dump channel includes CD19 and CD14 antibodies.
