# Supplementary material for: Dengue Mosaic Vaccines Enhance Cellular Immunity and Expand the Breadth of Neutralizing Antibody Against All Four Serotypes of Dengue Viruses in Mice
Source: Front Immunol. 2019 Jun 20;10:1429. doi: 10.3389/fimmu.2019.01429 (PMC6596366; doi:10.3389/fimmu.2019.01429)
Supplement: Supplementary file 4 [file Data_Sheet_3.PDF]

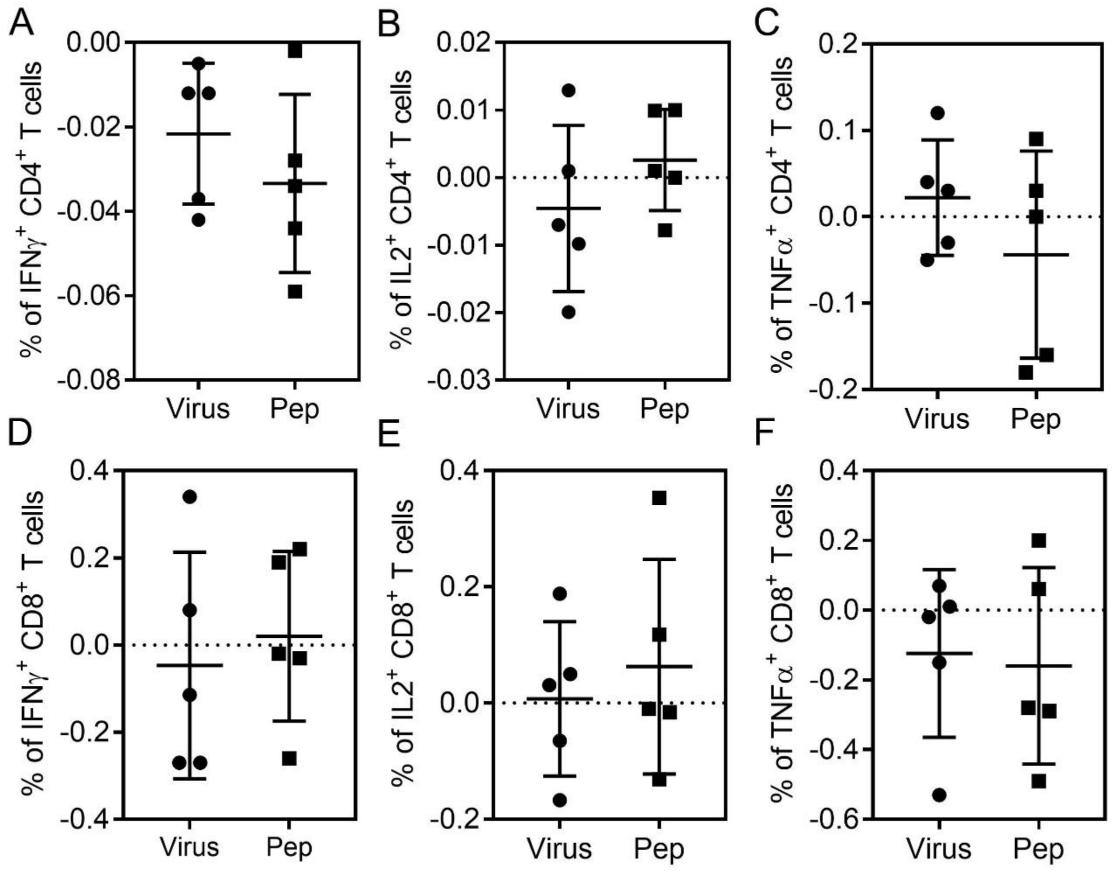

Supplement Figure 3. The cellular response background from mock control.

The cellular immune responses from control mock (PBS) immunized mice (3 injections with 2 weeks interval). In the ICS assay, we used mixed virus pool (consist of DENV1, DENV2, DENV3, and DENV4 virus), peptide pool and media control to stimulate the splenocytes, the data presented was normalized by subtracting the media control.
