# Supplementary material for: Dengue Mosaic Vaccines Enhance Cellular Immunity and Expand the Breadth of Neutralizing Antibody Against All Four Serotypes of Dengue Viruses in Mice
Source: Front Immunol. 2019 Jun 20;10:1429. doi: 10.3389/fimmu.2019.01429 (PMC6596366; doi:10.3389/fimmu.2019.01429)
Supplement: Supplementary file 2 [file Data_Sheet_1.PDF]

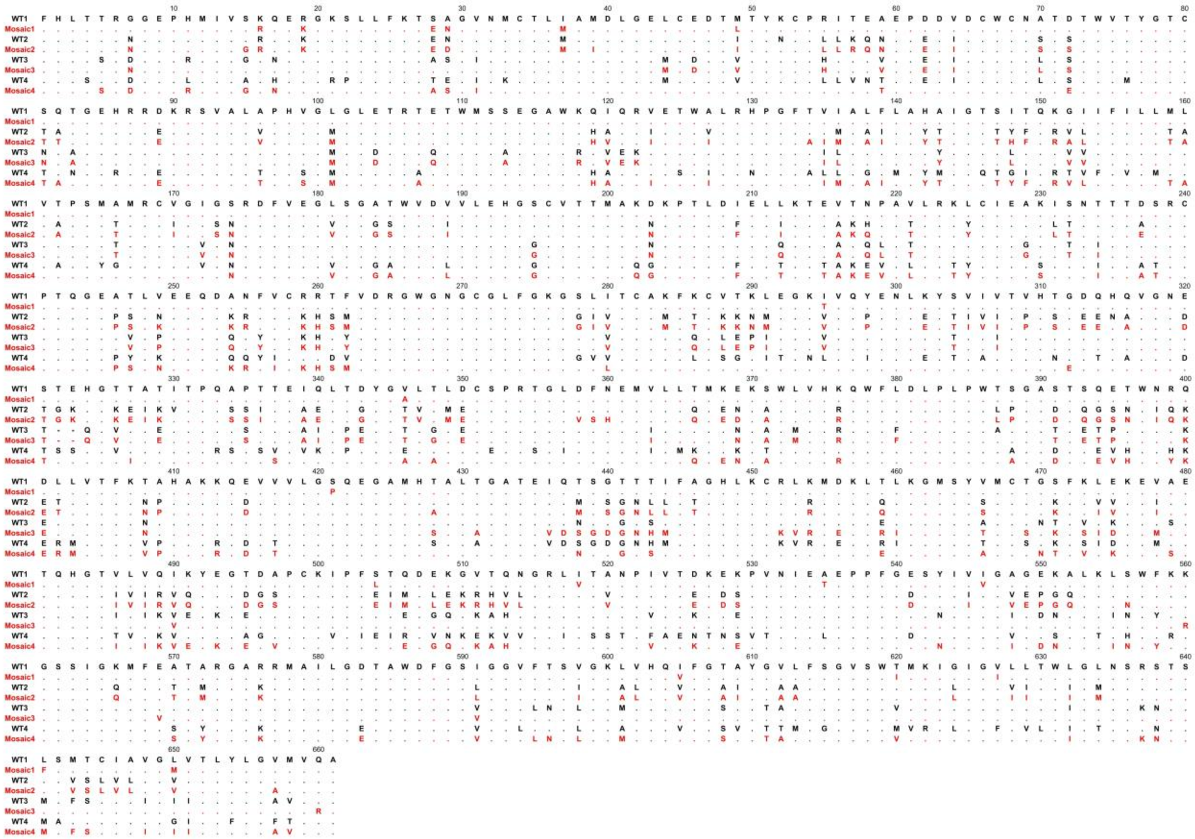

Supplement Figure 1. Sequence alignment and comparison.

Alignments of amino acid sequences of mosaic and wild type sequences. The first sequence (WT1) is considered as "template" sequence in this analysis. The identical amino acid from other sequences are shown as dot (.), otherwise the different amino acids are presented by the abbreviation of substituted amino acid.
